# Acceptability of workplace choice architecture modification for healthy behaviours

**DOI:** 10.1186/s12889-023-17331-x

**Published:** 2023-12-07

**Authors:** Eeva Rantala, Saara Vanhatalo, Federico J. A. Perez-Cueto, Jussi Pihlajamäki, Kaisa Poutanen, Leila Karhunen, Pilvikki Absetz

**Affiliations:** 1 Institute of Public Health and Clinical Nutrition, University of Eastern Finland, 70211 Kuopio, Finland; 2 VTT Technical Research Centre of Finland, 02044 Espoo, Finland; 3 VTT Technical Research Centre of Finland, Kuopio, 70211 Finland; 4 Finnish Institute for Health and Welfare (THL), 00271 Helsinki, Finland; 5 Department of Food, Nutrition and Culinary Science, Umeå University, 901 87 Umeå, Sweden; 6 Department of Medicine, Endocrinology and Clinical Nutrition, Kuopio University Hospital, 70029 KYS Kuopio, Finland; 7 Faculty of Social Sciences, Tampere University, 33520 Tampere, Finland

**Keywords:** Acceptability, Choice architecture, Nudge, Workplace, Health promotion, Prevention, Type 2 diabetes

## Abstract

**Background:**

Altering the choice architecture of decision contexts can assist behaviour change, but the acceptability of this approach has sparked debate. Considering hypothetical interventions, people generally welcome the approach for promoting health, but little evidence exists on acceptance in the real world. Furthermore, research has yet to explore the implementers’ perspective, acknowledging the multidimensionality of the acceptability construct. Addressing these knowledge gaps, this study evaluated the acceptability of a quasi-experimental implementation-effectiveness trial that modified the worksite choice architecture for healthy eating and daily physical activity.

**Methods:**

Fifty-three worksites participated in the 12-month intervention and implemented altogether 23 choice architecture strategies (Mdn 3/site), including point-of-choice prompts and changes to choice availability or accessibility. Retrospective acceptability evaluation built on deductive qualitative content analysis of implementer interviews (*n* = 65) and quantitative analysis of an employee questionnaire (*n* = 1124). Qualitative analysis examined implementers’ thoughts and observations of the intervention and its implementation, considering six domains of the Theoretical Framework of Acceptability: ethicality, affective attitude, burden, intervention coherence, opportunity costs, and perceived effectiveness. Quantitative analysis examined employees’ acceptance (7-point Likert scale) of eight specific intervention strategies using Friedman test and mixed-effects logistic regression.

**Results:**

Implementers considered the choice architecture approach ethical for workplace health promotion, reported mostly positive affective attitudes to and little burden because of the intervention. Intervention coherence supported acceptance through increased interest in implementation, whereas low perceived utility and high intensity of implementation reduced cost acceptance. Perceived effectiveness was mixed and varied along factors related to the implementer, social/physical work environment, employer, and employee. Employees showed overall high acceptance of evaluated strategies (Mdn 7, IQR 6.4–7), though strategies replacing unhealthy foods with healthier alternatives appeared less supported than providing information or enhancing healthy option availability or accessibility (*p*-values < 0.02). Greater proportion of male employees per site predicted lower overall acceptance (OR 4.4, 95% CI 1.2–16.5).

**Conclusions:**

Work communities appear to approve workplace choice architecture interventions for healthy eating and physical activity, but numerous factors influence acceptance and warrant consideration in future interventions. The study contributes with a theory-based, multidimensional evaluation that considered the perspectives of implementers and influenced individuals across heterogeneous real-world settings.

**Supplementary Information:**

The online version contains supplementary material available at 10.1186/s12889-023-17331-x.

## Background

Altering the choice architecture—the way available options are presented in behavioural contexts—is a subtle approach to “nudge” healthy behaviours without bans or substantial changes to incentives [1, 2]. The approach exploits people’s sensitivity to contextual cues and tendency to invest little deliberation in everyday choices [3], such as those related to eating or daily physical activity. The approach is rooted in the dual-systems models that assume behaviour to result from the interplay of automatic and reflective processes [4], and in the evidence of cognitive biases and heuristics that may prevent rational behaviour [2, 5]. At the workplace, choice architectures conducive to healthy eating and physical activity can promote the wellbeing and health of the workforce, which benefits the employer and the society as well [6, 7].

Choice architecture interventions typically work by increasing the salience or attractiveness of healthy options, by reducing the effort required to choose such options, or by leveraging social norms [8]. Due to the subtleness of these interventions and ability to change behaviour without people being aware of their presence or influence on behaviour [9], the ethicality of the choice architecture approach has stimulated a lively debate [10, 11]. While choice architecture strategies in principle maintain targeted individuals’ freedom of choice, in practice this freedom is questionable as the strategies target contexts where people typically fail to deliberate on their actions and to follow their reasoned preferences [10]. Hence, the intentional use of choice architecture strategies calls for careful consideration and responsibility, including comprehensive acceptability evaluation. Such evaluation reveals the approval of interventions among deliverers and receivers and facilitates the detection of factors that may influence implementation and effectiveness, hence supporting the interpretation of study outcomes and the development of enhanced interventions [12, 13].

Research on the acceptability of choice architecture interventions for healthy eating or daily physical activity relies predominantly on surveys that have examined public opinions on hypothetical interventions [14–27]. In these studies, the portrayed sources behind interventions have often been policymakers [14–20, 24, 27] but rarely employers [26] or related actors such as catering services [14, 21, 22]. Few studies have measured people’s approval of interventions after they have experienced the interventions in the real world [28–31]. Acceptability has been evaluated from the perspective of influenced individuals [14–31], and evaluations have covered varying interventions, including ones that alter the availability [15, 20, 24, 27], visibility and accessibility [16–20, 23, 25, 26, 28, 29], or labelling of choice options [15–17, 19, 24–27], or that provide tips, leverage social norms, or encourage commitment [21, 22, 25]. Whether measured as the proportion of approving respondents or as the degree of respondents’ approval, study participants have expressed overall support for evaluated interventions [14–31]. Acceptance appears to depend on various factors, however, including the type [15–21, 24, 26, 27], perceived effectiveness [14, 15, 18, 19], and intention of interventions [14, 16, 17, 23].

Henceforth, research on the acceptability of choice architecture interventions could start shifting focus from the public acceptance of hypothetical interventions towards the evaluation of real-world implementations, because predicted responses to imagined scenarios may not translate to interventions actually encountered [11]. Workplaces, in turn, merit more attention because the majority of working age population spends a substantial part of their time at work, making workplaces a suitable setting for health-promoting choice architecture interventions. Acceptability evaluations could also broaden their scope from the perspective of influenced individuals to that of the implementers who determine how interventions materialise. Moreover, besides commonly measured overall approval or beliefs about intervention effectiveness [14–31], studies could evaluate also other dimensions of acceptability. Acceptability has been defined as a multi-faceted construct that reflects the extent to which intervention deliverers or receivers consider the intervention appropriate, based on anticipated or experienced cognitive and emotional responses to the intervention [13]. An accompanying framework, the Theoretical Framework of Acceptability (TFA), proposes seven key dimensions of acceptability: ethicality, affective attitude, burden, intervention coherence, opportunity costs, perceived effectiveness, and self-efficacy [13]. The framework has served the acceptability evaluation of various health-promotion programmes (e.g., [32, 33]), including choice architecture interventions [34].

To broaden our understanding of the acceptability of the choice architecture approach, we aimed to evaluate the acceptability of a choice architecture intervention for healthy eating and daily physical activity at the workplace. The work contributes with a theory-based, multidimensional evaluation that included the perspectives of implementers and influenced employees once they had experienced the intervention. Simultaneously, the work provides insights on the feasibility of upscaling a broad range of choice architecture strategies to heterogeneous worksites. Such insights are valuable, as the success in translating promising interventions from controlled behavioural labs [35] or realistic living labs [36] to real-world operations is not guaranteed [37].

## Methods

### Study design and setting

The acceptability evaluation built on data collected during a 12-month quasi-experimental hybrid type 2 implementation-effectiveness trial [38], “StopDia at Work”, that was conducted between 2017 and 2019 in natural settings at workplaces in three regions of Finland (Northern Savo, South Carelia, and Päijät-Häme) [39]. The intervention promoted healthy dietary choices and daily physical activity with subtle modifications to the worksite choice architecture. The intervention was a part of a larger type 2 diabetes prevention study “StopDia” (Trial registration: NCT03156478) [40, 41] that was reviewed by the research ethics committee of the hospital district of Northern Savo (statement number: 467/2016, date of approval: 3 January 2017). The employees of intervention sites received general information on the StopDia study and the collaboration between their workplace and the study. However, the employees were not disclosed the specific aim of the StopDia at Work-intervention to alter the worksite choice architecture to promote healthy behaviours. This non-disclosure was to avoid interfering with employees’ natural responses to the intervention.

### Participating organisations

Sixteen organisations from various fields participated in the intervention with altogether 53 distinct worksites that employed in total 5100 employees (M 43% men) (Table 1). Ten of the organisations represented private sector and six public sector. Four organisations had worksite cafeterias that were involved in the intervention.

**Table 1:** Characteristics of participating organisations

Region^a^	Organisation^a^	Field of operation	Types of sites	n Sites	n Employees^b^	% Men
A	O1	Retail	Grocery	5	360	21
A	O2	Metal industry	Factory	1	600	80
A	O3	Forest industry	Factory^c^	1	950	78
B	O4	Retail	Grocery	3	300	20
B	O5	Higher education	University building	5	370	34
B	O6	Municipality	Bureau	1	70	29
B	O7	Chemical industry	Factory^c^	1	400	75
C	O8	Farming	Farm	1	140	35
C	O9	Municipality	Bureau	1	80	39
C	O10	Municipality	Bureau, kindergarten	3	250	32
C	O11	Construction industry	Construction yard, office	5	180	91
C	O12	Healthcare	Hospital department^c^	20	490	46
C	O13	Food industry	Factory	1	250	70
C	O14	Retail	Grocery	3	320	18
C	O15	Municipality	Bureau^c^	1	300	20
C	O16	Welfare	Welfare services centre	1	40	5

^a^Geographical regions and organisations are indicated with codes due to data protection

^b^Approximate number of employees exposed to the intervention

^c^Worksite cafeterias involved in the intervention

### Intervention content and implementation

The content and implementation of the intervention were tailored to each worksite to fit local contexts (facilities, resources, and employees’ needs concerning diet and physical activity), as detailed elsewhere [39]. Following bilateral dialogues between the research team and the participating organisations, intervention strategies were selected individually for each site from the StopDia Toolkit for creating health-promoting worksite environments [39]. The toolkit comprised evidence-based strategies that either altered the availability of healthy and/or less healthy options or that redesigned the arrangement, properties, or presentation of already available opportunities. The content built on the nudge approach [1, 2], the dual-systems models [4], and frameworks that characterise diverse choice architecture interventions [42–44].

Each organisation had at least one member of their personnel involved in designing and delivering the intervention at their sites. While designing included the planning of the content and implementation of the intervention to the worksite, delivery included the launch of selected intervention strategies and sustaining them over the 12-month intervention. Depending on the organisation, the designers and the deliverers could be the same or different individuals. Either way, we consider both the designers and the deliverers the implementers of the intervention. The implementers could also change over the intervention year due to staff turnover at the participating organisations.

**Fig. 1:**
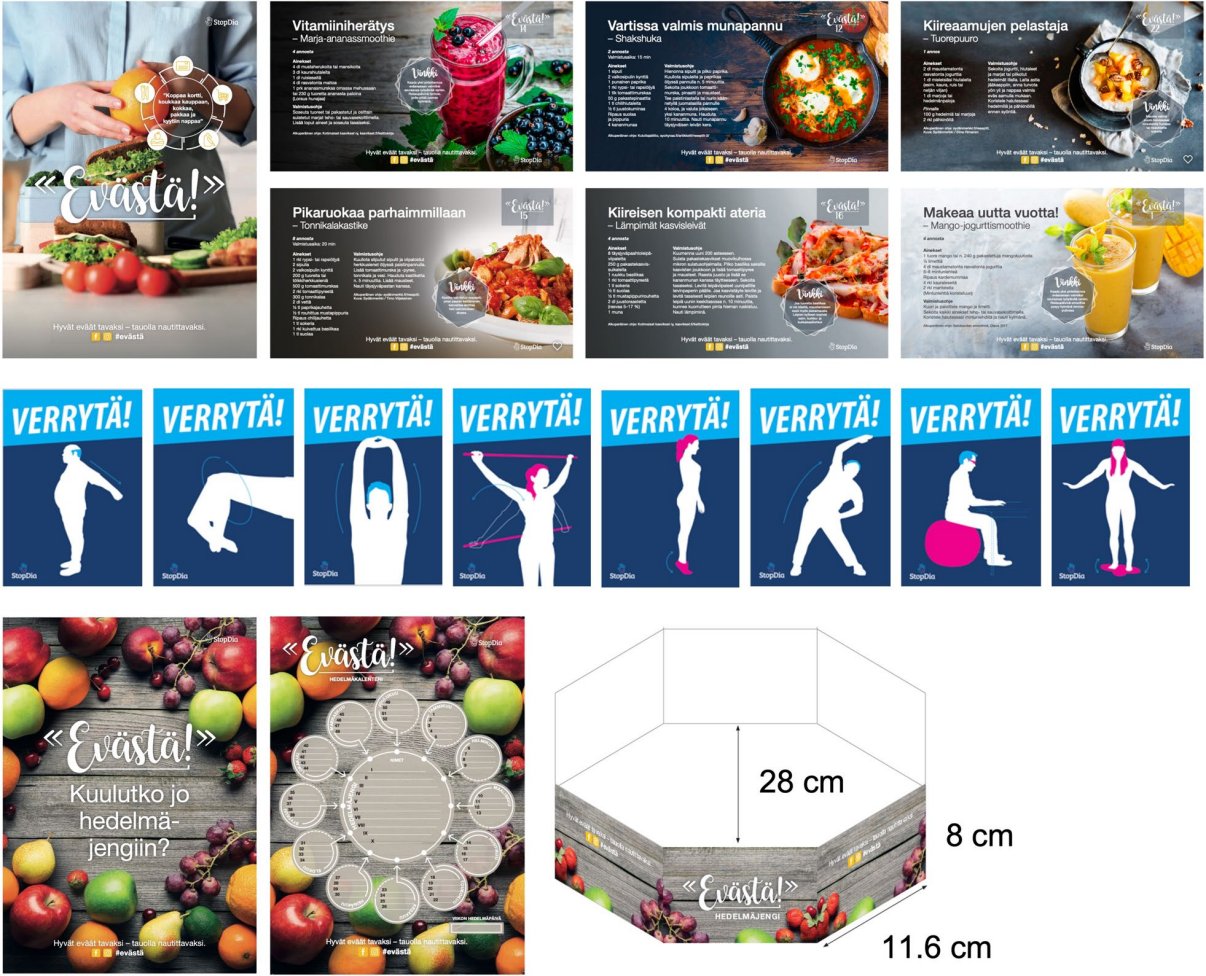
Examples of materials of most frequently implemented intervention strategies: #15 (top), #20 (middle), #16 (bottom). For descriptions of content, see Table 2

In total 23 choice architecture strategies were implemented across participating worksites, sixteen promoting healthy eating and seven physical activity (Table 2). The strategies applied numerous behaviour change mechanisms, including primes, prompts, and alterations to the availability, visibility, accessibility, convenience, or size of choice options. The median number of strategies intended to implement per site was three (range 2─14), a median of two (range 1─9) focusing on healthy eating and one (range 1─5) on physical activity. Except for one site, all sites also implemented at least one strategy. The three most often implemented strategies were a packed lunch recipe campaign (#15), a movement prompt strategy (#20), and a fruit crew-strategy (#16), respectively (Table 2, Fig. 1). Implementation settings comprised cafeterias, meetings, coffee rooms, common working areas, personal workstations, stairwells, and elevators. Participation was free of charge for the organisations, and the study provided intervention sites with materials for priming and prompting strategies, including posters, labels, and signs. If the sites chose to implement strategies that required other materials, such as exercise equipment or new food products to cafeterias, the sites were responsible for their procurement.

**Table 2:** Choice architecture strategies implemented and the number of organisations (sites) intending to implement each strategy

Target	Intervention strategy	Description	Ease of implementation	Setting	n^a^
Healthy eating					
Food provision	1. Enable healthy choices	Healthy food/beverage choices made available	Moderate	Meetings	6 (6)
	2. Widen selection	Greater variety of healthy food/beverage options available	Moderate	Cafeteria	4 (4)
	3. Replace with better alternatives	Less healthy options replaced with nutritionally better alternatives	Moderate	Meetings	1 (1)
	4. Increase visibility and proximity	Healthy options placed: (a) on visible spots, (b) at the beginning of the buffet, (c) closer to the chooser (e.g., in front row), and/or (d) in the middle of the tray, shelf, or showcase	Demanding	Cafeteria	4 (4)
	5. Decrease visibility and proximity	Less healthy options placed: (a) on less visible spots, (b) at the end of the buffet, (c) further away from the chooser (e.g., in back row), and/or (d) on the edge of the tray, shelf, or showcase	Demanding	Cafeteria	4 (4)
	6. Increase convenience	Fruit and vegetables served ready to eat (i.e., washed, peeled, cut into pieces)	Demanding	Meetings	1 (1)
	7. Increase perceived variety	Salad components served from individual containers	Moderate	Cafeteria	1 (1)
	8. Use smaller serving dishes	Less healthy options served from smaller serving dishes	Moderate	Cafeteria	1 (1)
	9. Use smaller serving utensils	Less healthy options served with smaller tongs and spoons	Moderate	Cafeteria	1 (1)
	10. Use smaller serving sizes	Less healthy options served in smaller sizes	Moderate	Meetings	2 (2)
	11. One plate-policy at lunch	Separate bread/salad plates moved out of sight to facilitate choosing one large plate and composing a meal according to the plate model (i.e., 1/2 vegetables, 1/4 protein, and 1/4 carbohydrates)	Easy	Cafeteria	1 (1)
	12. Point-of-choice prompts	Healthy options indicated with the Heart symbol^b^ on menus/at the point of choice	Demanding	Cafeteria	4 (4)
	13. Prime for better choices	“Follow the heart”-posters^b^ at cafeteria entrance/at the beginning of the buffet to facilitate noticing and choosing options carrying the Heart symbol	Easy	Cafeteria	4 (4)
Drinking water	14. Facilitate and remind of drinking water	Personal, reusable water bottles provided for employees	Easy	Personal workstation	2 (6)
Packed lunches and snacks	15. Encourage smart packed lunches	A year-long recipe campaign with temptingly named, visually attractive, seasonal, and healthy packed lunch recipes promoted at worksite coffee rooms, via electronic channels (info-screens, company intranet, newsletters), and on social media (Facebook, Instagram). The recipes covered various meal options (warm courses, salads, smoothies, sandwiches), included traditional Finnish dishes and dishes from around the world, and emphasised appealing sensory properties or ease of preparation. Campaign materials included one recipe for each week of the year, a poster, and a cardboard stand for printed recipe cards (Fig. 1)^b^. Campaign slogan encouraged to form a habit of enjoying good food during breaks and featured a rhyme that prompted to pick up a recipe, stop by the store, and prepare, pack, and grab the meal	Moderate	Coffee rooms	16 (53)
	16. Encourage provision of fruit at work	The promotion and provision of the “Fruit Crew”-starter set for forming fruit circles whose members take turns to organise fruit serving at work. The kit included a poster that asked: “Already a member of the fruit crew?”, an instruction and enrolment form, and a recyclable basket for fruit (Fig. 1)^b^	Easy	Coffee rooms	9 (37)
Physical activity					
Time spent sitting	17. Enable active sitting	Introduction of balance cushions or wobble stools	Easy	Common environments	1 (1)
Stair use	18. Enhance stairwell visibility	Footprints on the floor leading to stairs from the elevator	Easy	Elevator, stairs	2 (2)
	19. Prompt choosing the stairs	StopDia logo (a stop hand-sign with a heart on the palm)^b^ placed on elevator doors, next to elevator call buttons, or in their immediacy	Easy	Elevator	3 (6)
Movement breaks	20. Prompt context-specific movement	Posters encouraging to “flex” or “loosen up” and depicting simple movements suitable to be performed, e.g., by the copy machine, microwave, coffee maker, or bathroom (Fig. 1)^b^	Easy	Common environments	16 (53)
	21. Enable movement with exercise equipment	Light exercise equipment made available, e.g., gym sticks, balance boards, or hanging bars	Easy	Common environments	7 (10)
	22. Increase visibility and proximity of exercise equipment	Available exercise equipment placed on salient spots suitable for short exercise breaks, e.g., by the copy machine, microwave, or coffee maker	Moderate	Common environments	7 (14)
	23.Prompt movement with automatic reminders	Computer-based break exercise application provided for employees	Easy	Personal workstation	1 (2)

Healthy foods/beverages were defined as products, meals, and recipes that met the product category-specific nutrition criteria of the Heart symbol—the nutrition label of the Finnish Heart Association and the Finnish Diabetes Association [45], as well as energy-free beverages

^a^Total number of organisations was 16 (total number of sites was 53)

^b^For images and additional details of the materials, see the Supplementary Material S1 of [39]

We defined the ease of implementation of each intervention strategy on a three-point scale (easy, moderate, demanding) based on discussion within the research team (Table 2) [39]. The classification reflected the amount of knowledge and effort required from the implementer to sustain the strategy after its launch. Easy strategies required little specialised knowledge, and besides occasional check-ups, no actions after launch. Examples included laying out posters and introducing new equipment or furniture. Moderate strategies required some knowledge on correct implementation and light maintenance on a regular basis. Examples included maintaining exercise equipment in pre-defined places and running the packed lunch recipe campaign that required a weekly delivery of materials. Demanding strategies required more specialised knowledge on correct implementation and daily maintenance. Examples included the use of nutrition labels and the placement of healthy vs. unhealthy foods at worksite cafeterias. We judged ten of the employed strategies easy to sustain, nine moderate, and four demanding. The three most often implemented strategies fell under the categories easy and moderate.

### Data collection

#### Implementer perspective

For qualitative, implementer-level evaluation of acceptability, we collected data with semi-structured interviews from the implementers of participating organisations (Additional file 1). Email and text messages received from the implementers complemented the interview data. As applicable, we portray the qualitative data collection and analysis following the checklist of the consolidated criteria for reporting qualitative studies (COREQ) [46].

The first two authors (E.R., female MSc student in nutrition, and S.V., female PhD in nutrition) interviewed the implementers twice along the intervention. Majoring in clinical nutrition, both interviewers had received training in interviewing people. The interviewers had become acquainted with 55% of the implementers over the recruitment of participating organisations and the development and launch of the intervention. The implementers were familiar with the purpose of the intervention and the interviewers’ institutional affiliations, job titles, and roles in the study. In a healthcare organisation (O12) with 20 intervention sites, sites with patients were not accessible to externals. Hence, the head implementer of this organisation (female HR assistant) conducted the data collection at these sites with instructions from the research team.

In total 65 implementers contributed to the acceptability evaluation, at least one from each participating organisation (Table 3). The implementers represented diverse occupational groups and both management- and employee-level personnel. Of the implementers, 49% had been involved in designing the content and implementation of the intervention to their sites (i.e., "designers”), and 28% had jobs that essentially focused on the promotion of employee wellbeing and health (i.e., “health promoters”). The health promoters comprised HR, occupational wellbeing, and work ability personnel, and health and safety representatives. Without a couple of exceptions, the health promoters were also designers. The proportion of implementers without the designer’s and health promoter’s role (i.e., “other implementers”, such as assistants and catering personnel) was 48%. Information on gender was available for 51/65 implementers, and of these, 40 were female. Unknown gender concerned the implementers who were interviewed by the head implementer of O12.

**Table 3:** Number and work substance of implementers who contributed to the acceptability evaluation

Organisation	Total^a^	Designers^b^	Health promoters^c^	Other implementers^d^	Substance of work
O1	1	1	1	0	HR, communication
O2	1	1	1	0	Occupational wellbeing
O3	4	2	2	1	Work ability, communication, supervision of employees’ interests regarding employment, physical activity coaching
O4	1	1	1	0	Occupational wellbeing
O5	5	2	2	3	HR, assistance
O6	1	1	1	0	Occupational wellbeing
O7	7	4	2	2	HR, production, catering
O8	1	1	1	0	HR, finance
O9	2	2	2	0	HR, work ability
O10	6	1	1	5	HR, finance, building security and maintenance, early childhood education, administrative assistance, catering
O11	6	5	0	1	Housing construction, housekeeping
O12	18	3	1	15	HR, catering, healthcare
O13	1	1	1	0	HR
O14	5	3	0	2	Management, sales
O15	3	3	2	0	HR, health and safety, catering
O16	3	1	0	2	Management, administrative assistance, social work
Total	65	32	18	31	

^a^Total number does not equal the sum of designers, health promoters, and other implementers because most health promoters were also designers

^b^Involved in intervention design

^c^Substance of work focused on the promotion of employee wellbeing and health

^d^Implementers who were not designers nor health promoters

The first interview round took place halfway through the intervention approximately at month six and the second round at the end of the intervention approximately at month twelve. The interviews were conducted in person at the intervention sites as part of follow-up visits for monitoring implementation. The median duration of the follow-up sessions was 60 min on the first round and 30 min on the second round. These sessions comprised the interview and an implementation quality assurance tour in the worksite environment. The interviews took place at meeting rooms or at the implementers’ personal workstations. In open and shared workspaces, personnel not involved in the interviews could be within earshot. If on-site visits were not feasible, the interviews were conducted via Skype for Business-online meeting tool (Microsoft Corp., Redmond, WA, USA), on the phone, or by email. The interviewers made notes during the interviews and typed the notes up after the interviews. The transcribed notes were not returned to the interviewees. The number of interviews per organisation and the number of interviewees per interview varied along the number of intervention sites and implementers each organisation had. Additionally, the interviewees of each organisation could vary from one time point to another, for example, due to staff turnover.

The interviews followed a semi-structured outline devised by the research team (E.R., S.V., K.P., L.K., P.A.). Besides the first two authors, the team included professors and senior lecturers with expertise in the fields of public health, nutrition, behavioural sciences, and implementation research. See Additional file 1 for English translations of the interview questions relevant to the acceptability evaluation. The first interview round mapped the implementers’ views on the ethicality of the employer’s attempts to influence the employees’ health behaviour and enquired about the acceptability of the choice architecture approach in the promotion of healthy eating and physical activity among employees. Choice architecture interventions were portrayed to alter the worksite environment to subtly guide employees to health-promoting choices. In addition, the interviews asked about the implementers’ experiences of the implementation and about observed effects of the intervention. The second interview round collected complementary data on implementation and observed effects. Regarding the sites of O12 that were not accessible to externals, the head implementer toured the sites once after six months and collected experiences of the intervention and its implementation with an adapted interview outline.

#### Employee perspective

For quantitative, employee-level acceptability evaluation, we conducted a questionnaire at the end of the intervention among the employees of intervention sites. The employees were invited to answer a short questionnaire either online via the Questback®-tool (www.questback.com) or with paper and pen, depending on which was feasible for the worksite. A cover letter informed that the questionnaire was a part of the StopDia study and aimed to explore employees’ thoughts on workplace wellbeing promotion. Completing the questionnaire was voluntary and anonymous, took approximately five minutes, and required no identifiable information.

The questionnaire included nine acceptability-related items. One item asked whether the respondent finds acceptable (yes/no) that the employer seeks to influence the employees’ dietary and physical activity patterns with the aim of promoting the employees’ wellbeing. Eight items were informed by measures used in prior acceptability evaluations [20, 21, 24] and asked the respondent to rate on a seven-point Likert scale (completely disapprove—completely approve) the acceptability of eight specific choice architecture strategies that would be implemented by the employer (for strategy descriptions, see results). Additionally, the respondent could choose an opt-out option “I cannot say”. The rated strategies employed four types of behaviour change mechanisms: 1) the provision of information/tips, 2) point-of-choice prompts, 3) alterations to the availability of healthy options, and 4) enhancements to the visibility and accessibility of healthy options. The strategies resembled those most frequently implemented in the StopDia at Work-intervention.

The questionnaire also asked the respondent’s predominant quality of work (physical vs. less physical), typical meal location (worksite cafeteria vs. else), and whether the respondent wished for support for healthy eating or physical activity from the employer. Data on the percentage of male employees per intervention site during the intervention year were received from the implementers (Table 1).

### Analyses

#### Implementer perspective

The implementer-level acceptability evaluation applied deductive qualitative content analysis [47], building the coding framework upon the domains of the Theoretical Framework of Acceptability (TFA): ethicality, affective attitude, burden, intervention coherence, opportunity costs, perceived effectiveness, and self-efficacy [13]. The TFA defines ethicality as the extent to which the intervention fits an individual’s value system; affective attitude as how an individual feels about the intervention; burden as the perceived amount of effort that is required to participate in the intervention; intervention coherence as the extent to which an individual understands the intervention and how it works; opportunity costs as the extent to which benefits, profits, or values must be given up to engage in the intervention; perceived effectiveness as the extent to which the intervention is perceived as likely to achieve its purpose; and self-efficacy as the participants’ confidence that they can perform the behaviours required to participate in the intervention [13].

The analysis built on pooled data from the two interview rounds. Comparison between the two rounds was not meaningful, as the samples of interviewees and discussed topics were not identical across the two time points. The first author (E.R.) familiarised herself with the interview data through reading and rereading, simultaneously coding the data according to the domains of the TFA. The coding was not mutually exclusive, meaning that the same comment could relate to multiple themes and hence receive several codes. As the analysis identified no content related to the self-efficacy domain, we removed the domain from the coding framework.

We promoted the validity and reliability of the coding through a peer-checking process common in qualitative research [48, 49]. The first author reviewed quotes from the interview data against suggested coding with three other authors (S.V., L.K., P.A.), and the four authors refined and agreed on the coding. For data management and analysis, we used NVivo R1 (QRS International) and Microsoft Excel® 2016 (Redmond, WA, USA). As the period between data collection and analysis was substantial, contacts were lost to many interviewees and asking the interviewees to provide feedback on the results was not feasible.

#### Employee perspective

The employee-level acceptability evaluation examined the questionnaire data with descriptive statistics (frequencies/percentages, measures of central tendency and dispersion). Friedman test—the non-parametric alternative for repeated measures ANOVA—with Dunn-Bonferroni post hoc analysis for pairwise comparisons tested for differences in the distributions of acceptance across the eight specific choice architecture strategies rated. A non-parametric test was appropriate because the acceptance of the strategies proved non-normally distributed based on visual inspection of histograms and the Kolmogorov–Smirnov test of normality (*p*-values < 0.001). An overall acceptance score of the specific strategies was computed by averaging the ratings of respondents who rated all eight strategies. A mixed-effects logistic regression model with site-level random intercept explored associations between the overall acceptance score and relevant available site-level predictors: the proportion of male employees, respondents with physical work, respondents eating at the worksite cafeteria, and respondents hoping for support in healthy eating or physical activity (for details of the model, see Additional file 1). For the model, the acceptance score was transformed into a dichotomous variable, with scores below the 25^th^ percentile treated as the target category and scores at or above the 25^th^ percentile as the reference category. This cut-off point ensured both categories had sufficient sample sizes and variation in the predictors and the acceptance score. Statistical analyses were performed with IBM SPSS® Statistics 29.0 (IBM Corp., Armonk, NY, USA), considering *p*-values < 0.05 statistically significant.

In questionnaires completed with paper and pen, responses that fell between two options or that indicated multiple options were coded missing in the dichotomous yes/no-item (0.1% of total responses) and according to the lower rating in the scale items (0.1% of total). The overall percentage of missing data ranged from 0 to 0.9% across the questionnaire items. Opt-out responses (“I cannot say”) to the scale items were examined separate from the numeric responses.

## Results

### Implementer perspective

Acceptability-related findings drawn from the implementer interviews reflected six of the seven domains of the Theoretical Framework of Acceptability (TFA): ethicality, affective attitude, burden, intervention coherence, opportunity costs, and perceived effectiveness [13] (Table 4). The findings projected the implementers’ thoughts and observations on the content, implementation, and effectiveness of the StopDia at Work-intervention, as well as engagement in the promoted behaviours. The absence of the seventh TFA domain, self-efficacy (i.e., confidence in ability to participate in the intervention [13]), was unsurprising because choice architecture interventions are relatively simple to implement and typically encourage behaviours that require no advanced skills.

**Table 4:** Key findings of the implementer-level evaluation of acceptability

Domain	Definition^a^	Topic of implementer reports^b^	Key findings^b^
Ethicality	The extent to which the intervention fits the implementers’ values	Workplace health promotion in general or with the choice architecture approach	• Workplace health promotion benefits everyone and is acceptable with voluntary, positive, and encouraging interventions • The choice architecture approach is an ethical, gentle, and freedom-preserving way to encourage healthy choices
Affective attitudes	Feelings about the intervention	Intervention content and implementation	• Expressed attitudes were mainly positive and covered the choice architecture approach, implemented strategies, intervention materials, implementation, and the StopDia project • Packed lunch recipes drew some criticism • Perceived ineffectiveness reduced motivation for implementation
Burden	The perceived amount of effort that participation requires	Implementation, engagement in the promoted behaviours	• Once fallen into a routine, implementation felt effortless • Recipes could have been simpler
Intervention coherence	The extent to which the implementers understand the intervention and how it works	Implementation	• Understanding increased interest in implementation
Opportunity costs	The extent to which benefits, profits, or values must be given up due to the intervention	Financial investments required for intervention materials and implementation	• Low perceived utility of the intervention and high intensity of implementation reduced cost acceptance
Perceived effectiveness	The extent to which the implementers perceive the intervention likely to achieve its purpose	Observed effects or beliefs about the effectiveness of specific intervention strategies, general reflections on effectiveness	• Perceived effectiveness was mixed, clustering around positive and neutral observations • Facilitators for effectiveness involved an active implementer, supportive social and physical work environment, and employer-granted financial support for implementation • Suggested explanations for ineffectiveness included varying individual preferences, needs, and understanding of the intervention; and unsupportive circumstances at work

^a^Adapted from the Theoretical Framework of Acceptability [13]

^b^Unless otherwise specified, the topics of implementer reports and the key findings refer to the content, implementation, or perceived effectiveness of the StopDia at Work-intervention, or to engagement in the behaviours this intervention promoted

**Fig. 2:**
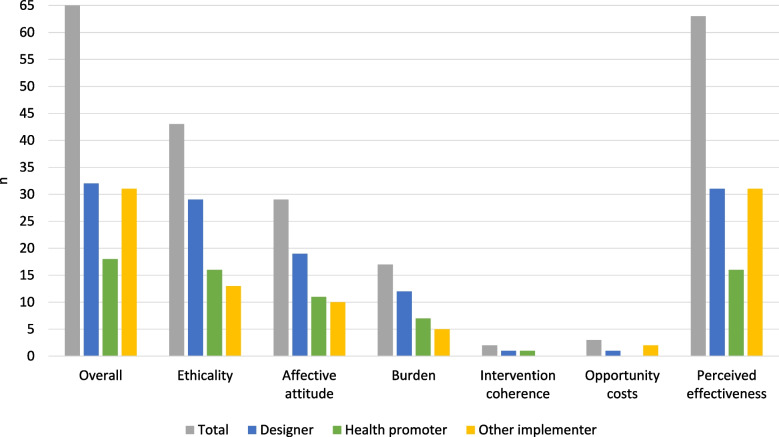
The number of implementers who contributed to the acceptability evaluation overall and by domain. Total does not equal the sum of designers, health promoters, and other implementers because most health promoters were also designers

The domains with the greatest number of contributing implementers were perceived effectiveness, ethicality, and affective attitude, respectively (Fig. 2). Among the implementers who contributed to each domain, the share of designers (i.e., implementers involved in the designing phase of the intervention), health promoters (i.e., implementers whose work focused on the promotion of employee wellbeing and health), and other implementers (i.e., individuals not involved in designing nor health promotion) varied across domains.

The following sections portray our findings related to each included domain. In accordance with the coding used in Tables 1 and 3, we indicate the organisations whose implementers contributed to each finding with the identifiers O1–16. Where feasible, we refer to specific intervention strategies to which the implementers referred using the numbering (#) presented in Table 2.

#### Ethicality

Regarding the legitimacy of workplace health promotion in general, implementers across participating organisations (O1–16) and implementer groups (27 designers, 16 health promoters, 11 other implementers) considered acceptable that the employer attempts to influence the employees’ health behaviour to promote the employees’ wellbeing and health. The employer’s efforts to support healthy behaviours were considered to benefit everyone, the employer and the employee (O11, O13), as well as the society (O15). Omitting such efforts could at worst lead to dismissals if employees were no longer able to work (O15), and societal resources would not suffice to cover health care costs (O15). Another argument was that when hiring personnel, employers have the right to expect employees to stay able to work (O10). Yet, some implementers noted that the line between acceptable and non-acceptable attempts to influence employees’ health behaviour is fine (O3, O16); while some greet health promotion measures with enthusiasm, some find them fraught (O14).

When the implementers were asked to specify the ways in which the employer may attempt to influence the employees’ health behaviour, they characterised acceptable attempts as positive (O3, O5, O15) and encouraging (O2, O8, O10, O14–15) measures that provide voluntary opportunities (O1─16). Mentioned opportunities could target the worksite environment with various choice architecture strategies or rely on the provision of information, incentives, or work arrangements.

Choice architecture interventions were considered ethical across organisations (O1–O16) and implementer groups (27 designers, 16 health promoters, 11 other implementers), mainly because they maintain employees’ freedom of choice (O4–6, O10, O12)—or as one implementer (O9) put it: “because they do not force employees to do anything. The environment just offers opportunities, and employees may choose whether to follow the cues”. Mentioned opportunities through which the worksite environment could promote healthy behaviours included ergonomic furniture such as height-adjustable desks (O10, O16); the availability, arrangement, and presentation of healthy foods at worksite cafeterias and meetings (O1, O7, O9, O12), as well as facilities and equipment for physical activity (O10). Implementers also supported the way in which choice architecture interventions can create contexts that “wake up” (O1) without being too “flagrant” and hence “pushing” (O11), and how these contexts can facilitate choices that experts have evaluated beneficial for health (O13). One implementer (O15) expressed their support for choice architecture strategies by noting: “The living environment influences behaviour anyway, so we can just as well build an environment that guides to healthy choices”.

#### Affective attitudes

##### Positive affective attitudes

Positive affective attitudes were expressed by 26 implementers (18 designers, 11 health promoters, 8 other implementers), at least one from each participating organisation. Positive attitudes focused on the choice architecture approach, implemented intervention strategies, intervention materials, intervention implementation, and the StopDia project as a whole. The choice architecture approach was well received, as implementers described the approach “very nice”, “good”, “friendly”, and/or “sensitive” (O2, O4). Regarding implemented strategies and materials, implementers reported positive attitudes towards strategies targeting the food provision at worksite cafeterias (Table 2: strategies #1, 2, 4, 5, 8, 9, 12, 13; organisation O3), strategies targeting packed lunches (#15; O3, O7, O9, O10–13, O15–16) and snacks (#16; O12, O14) in coffee rooms, and strategies encouraging physical activity (#18─20; O1, O3, O9, O16). In one organisation (O3), implementers described strategies implemented at the worksite cafeteria “brilliant” and “the best offering of the project” and found the changed look of the cafeteria “refreshing”. These implementers were satisfied also with the materials provided for other implemented strategies, which encouraged smart packed lunches (#15), stair use (#18─19), and context-specific movement (#20): “The materials were good, clear, and easily accessible, and instructions were good. Particularly the packed lunch recipes were good material”*.* The implementer of another organisation (O13) was content with the tone of the packed lunch recipes (#15): “The recipe cards do not feel pushing or imposing; their health-promoting message does not come across negatively”*.* In a couple of organisations (O1, O8), implementers found that the strategies implemented (#1, 10, 15, 20) suited their organisation and supported prior occupational wellbeing measures.

As for the implementation, several implementers were gladly involved (O10, O12), particularly after the implementation had formed into a routine (O10). Additionally, implementers welcomed the opportunities for breaks and physical activity that their implementation tasks afforded (O7–8, O11). One implementer (O5) was unable to suggest any improvements to the implementation process. In addition, implementers were content with the 12-month duration of the intervention (O10, O14). Regarding the StopDia project, several implementers expressed their satisfaction and found the project and its cause good, positive, and/or useful (O3–4, O6, O8, O14).

##### Critical affective attitudes

More critical attitudes came from altogether eleven implementers (6 designers, 1 health promoter, 5 other implementers) who represented five organisations. These attitudes focused on the packed lunch recipe strategy (Table 2, Fig. 1: #15), including its materials and their implementation. Regarding the materials, comments showed the variability of inter- and intra-individual food preferences. On one hand, implementers could hope for more basic recipes that include common, local ingredients (O8, O16). On the other hand, they could state that the recipes appeared tasteless and required “tuning”, for example, with added fat or seasoning (O16, O10). In terms of implementation, one implementer (O10) struggled finding motivation in the beginning of the intervention: “At first, I didn’t find motivating to change the recipe cards because the job felt an additional, unconnected work task that required remembering”. However, once the task formed into a routine, motivation increased. Related to perceived effectiveness, implementers at three sites (O11, O14) lost their motivation to sustain the recipe strategy due to perceived ineffectiveness.

#### Burden

Burden-related comments referred to implementation and engagement in the promoted behaviours. Fifteen implementers (11 designers, 6 health promoters, 4 other implementers) from nine organisations (O1, O5–6, O9–12, O14, O16) considered the implementation to cause little or no burden, portraying the implementation “easy”, “simple”, “natural”, and/or “effortless”. A couple of implementers, however, experienced the packed lunch recipe strategy (Table 2: #15) more burdensome. According to our categorisation, this strategy was moderate to sustain, defined as requiring some knowledge on correct implementation and light maintenance on a regular basis. One of the implementers (O10, other implementer) noted that remembering to update the recipe materials weekly was challenging at first. This burden reduced over time, however, as the implementation “fell into a routine”. The other implementer (O1, designer and health promoter) found the recipe strategy too burdening to sustain, as regards uploading the recipes on info screens and timing their display. Regarding the engagement in the promoted behaviour, two implementers (O8, designer and health promoter; O11, designer) felt that the packed lunch recipes should have been less burdensome, meaning “simpler” and “quicker” to prepare.

#### Intervention coherence

Comments that reflected intervention coherence were related to implementation. One implementer (O12, designer and health promoter) portrayed that understanding the rationale behind the intervention motivated them to implement: “The study woke me to think of type 2 diabetes and that I wouldn’t want to get it. That raised my interest in the choice architecture approach as well”. Via personal interest, this comment draws a link between intervention coherence and affective attitudes. Another implementer (O4, designer and health promoter) had an opposite experience. This implementer participated in intervention design but delegated the responsibility of delivery to site managers via email instructions. The implementation in this organisation proved less successful. The implementer pondered that the lack of understanding could explain the poor performance: “the site managers might not see the connection between health promotion activities, diabetes, and, for example, absence from work”.

#### Opportunity costs

Cost-related remarks concerned the financial investments that intervention materials and their implementation required. Two implementers (O12, other implementers) criticised the public funding and efforts invested in the packed lunch recipe strategy (Table 2: #15). These comments reflected frustration with the labour policy that the ruling government had implemented. One implementer said: “I don’t really understand why they (i.e., the recipe cards) are like this (i.e., printed). Wouldn’t electronic materials be more contemporary? The cards have consumed plenty of money and printing materials. I admit that the past years’ cuts in hourly wages nag me while I change the cards and sign the checklist—that this can be afforded”. The other implementer thought: “taxpayers’ money should not be spent on this (i.e., the recipe materials) but on something more important”.

At one site (O14) that chose to implement the fruit crew strategy (#16) by treating employees with unlimited fruit daily, costs appeared too high for sustained implementation. Interestingly, at another site of the same organisation, no cost-related issues emerged once the same strategy was delivered with less intensive implementation; by providing each employee one fruit on two days of the week.

#### Perceived effectiveness

**Fig. 3:**
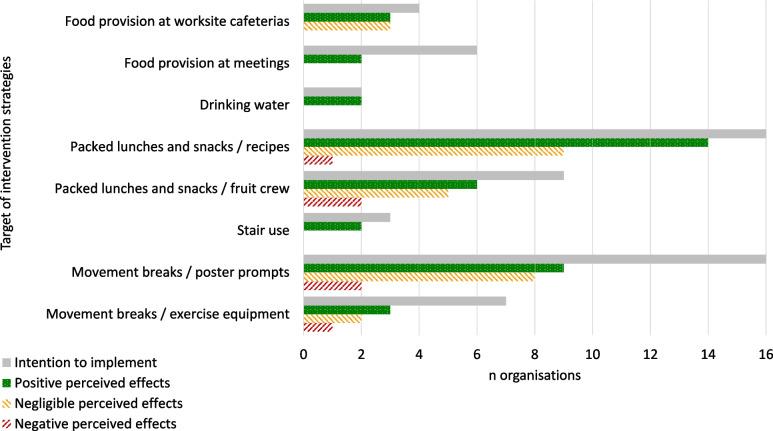
Strategy-specific perceived effects reported from organisations that intended to implement corresponding strategies. Total number of participating organisations 16. Coding is not mutually exclusive

Perceived effectiveness was overall mixed, clustering around positive and negligible findings and varying both between and within strategies, organisations, and implementers (designers, health promoters, other implementers). Reports of perceived effectiveness consisted mostly of implementers’ observations of effects that specific intervention strategies had elicited in themselves or in the rest of the personnel of their worksites. These observations concerned strategies that targeted the food provision at worksite cafeterias or meetings, drinking water, packed lunches and snacks, stair use, and movement breaks (Fig. 3, Table 5). Across the strategy-specific observations, positive perceived effects were reported from 15, negligible from 12, and negative from four organisations. In addition, the comments of a few implementers reflected beliefs rather than actual observations, and some implementers discussed effectiveness more generally.

**Table 5:** Examples of positive ( +), negligible ( ~), and negative ( −) perceived effects of specific intervention strategies

Target and corresponding intervention strategies	Examples of perceived effects (O = organisation, # = strategies implemented)
Healthy eating	
Food provision at worksite cafeterias 2. Widen selection 4. Increase visibility and proximity 5. Decrease visibility and proximity 7. Increase perceived variety 8. Use smaller serving dishes 9. Use smaller serving utensils 11. One plate-policy 12. Point-of-choice prompts 13. Prime for better choices	+ Changes in the cafeteria were eye opening; how small changes influenced behaviour. Implementers perceived that the intervention had resulted in lighter eating (O3: #2, 4, 5, 8, 9, 12, 13) + Consumption of salads and fruit increased, consumption of main courses and carbohydrate accompaniments (i.e., mashed/boiled potatoes, rice, pasta) decreased. The implementers also noticed that they themselves started to consume more salad in the cafeteria (O7: #2, 4, 5, 11 − 13) + / ~ Some customers noticed the point-of-choice Heart symbols and chose corresponding foods, some did not (O12: #2, 4, 5, 7, 12, 13) ~ No observed effects on customers’ food choices (O15: #2, 4, 5, 12, 13) ~ No observed effects on breakfast porridge consumption (O7: #2, 4, 5, 11 − 13)
Food provision at meetings 1. Enable healthy choices 3. Replace with better alternatives 6. Increase convenience 10. Use smaller serving sizes	+ Meeting organisers increased orders of fruit and decreased orders of sweet buns (O10: #1; O11: #1, 3, 6) + Serving fruit ready to eat (e.g., peeled) reduced food waste (O11) + Employees gave positive feedback on fruit served at meetings (O11). [A positive change in attitudes over the intervention]
Drinking water 14. Facilitate and remind of drinking water	+ Water bottles were used (O5, O9)
Packed lunches and snacks 15. Encourage smart packed lunches (the packed lunch recipe campaign)	+ At least some employees/implementers took recipes (O1–2, O6–12, O14–16) + At least some employees/implementers tried recipes (O2, O5–6, O9–10, O12–13, O16) + The employees were allowed to try a recipe at work during working hours, and the prepared food was served at the worksite’s weekly brunch (O16) + More recipes were taken when presented and handed out to employees personally (O10) + If one employee reviewed and commented on a recipe, other employees could take the recipe as well (O11) + Employees looked forward to upcoming recipes (O7, O12–13, O15) and asked when they appear (O12–13) + Employees who did not speak Finnish as their first language tried to translate the recipes in English (O8) ~ Overall, few recipe cards were taken (O1, O6, O9–12, O14–16) ~ Recipes were taken but not prepared (O14, O16) ~ Recipes could remain unused if they included ingredients not available at home or ingredients not usually used in home cooking (O11, O14, O16) − Posters were torn down over the intervention year (O2)
Packed lunches and snacks 16. Encourage the provision of fruit at work (the Fruit Crew-strategy)	+ The strategy was in active use at least in some coffee rooms or some parts of the worksite, with the costs of provided fruit covered by the employees (O9, O15) or by the employer (O14) + The strategy was in use in coffee rooms where the community spirit was high and where the employees actively organised events and common activities (O15) + Employees occasionally brought fruit for everyone to enjoy, e.g., during the harvest season (O10) or Christmas (O16) + /– In the beginning of the intervention, employees in the day shift took so many fruit that none were left for employees in the evening shift. Once instructions were clarified (one fruit/employee), the strategy began to work, and the fruit sufficed for everyone (O14) ~ The strategy was not in active use (O1, O10, O12, O15, O16) ~ No fruit crews were formed because the employees ate plenty of fruit anyway and found the strategy useless (O12) ~ The fruit basket of the “Fruit Crew”-starter set was used for something else than for serving fruit, e.g., for keeping pens (O15, O12)
Physical activity	
Stair use 18. Enhance stairwell visibility 19. Prompt choosing the stairs	+ Implementers perceived increased stair use (O3, O6)
Movement breaks 20. Prompt context-specific movement	+ Movements were performed (O5–12, O14) ~ Implementers saw no one perform any movements (O10–14) ~ Implementers themselves performed no movements although the posters were in sight (O12, O16) − In the beginning of the intervention, the posters were removed from bathrooms (O7)
Movement breaks 21. Enable movement with exercise equipment 22. Increase visibility and proximity of exercise equipment	+ At least some employees used at least some of the available equipment (O3, O9, O14) + The equipment tended to disappear/travel away from its intended place, indicating potential use (O9) + / ~ Balance cushions on seats shared opinions; some used them, some not (O9) ~ Equipment was not used (O14) − For some, sitting on balance cushions caused nausea (O9)

Positive perceived effects of eating-related strategies appeared in increased availability and consumption of nutritionally high-quality foods, such as vegetables and fruit at worksite cafeterias, meetings, or coffee rooms (Table 5). Further positive observations included employees’ interest in and the use of the promoted packed lunch recipes, as well as the use of water bottles provided for employees. With strategies promoting daily physical activity, positive perceived effects emerged as increased movement and the use of stairs and available exercise equipment. Factors that accompanied positive observations were related to the implementer, the social and physical work environment, and the employer. Examples included the implementers’ initiative to present and deliver print intervention materials to employees personally (O10), positive behavioural examples set by colleagues (O11), high community spirit and active employees that were used to organising common activities (O15), the opportunity to use working hours and worksite facilities to prepare and enjoy packed lunches together with colleagues (O16), and the employer’s financial support for organising fruit provision in coffee rooms (O14).

Reports of negligible perceived effects were nearly as common as reports of positive perceived effects. In addition, perceptions of positive and negligible effects often coexisted, as implementers could observe positive effects in some employees or behaviours while negligible effects in other employees or behaviours. Regarding strategies targeting packed lunches and snacks (Table 2: #15–16), implementers reflected potential reasons for the mixed or negligible effects. Suggested explanations included employees’ varying needs for (O10, O12, O16) and understanding of (O1, O8) the strategies, varying food preferences (O9, O11), as well as large work communities and shift work that challenged the organisation of and engagement in common activities (O1, O12).

Negative perceived effects were rare and appeared in tearing down of posters (O2, O7), in hoarding of fruit that the employer provided (O14), and in reports of unpleasant feelings after the use of certain exercise equipment (O9). Some of these effects occurred only in the beginning of the intervention and disappeared through enhanced implementation and communication with the employees (O7, O14).

Besides actual observations, a few implementers expressed sceptical beliefs in the effectiveness of strategies promoting healthier eating. While one implementer (O10, other implementer) considered that “eating at work can hardly be influenced”, another (O15, designer) thought that strategies at the worksite cafeteria “won’t help if people have no motivation” and “matter little because people eat what they wish at home”. One implementer of a healthcare organisation (O12, other implementer) expected the packed lunch recipes to bear little effect: “I doubt the resulting health benefits are very significant. Particularly in hospitals people have so much nutrition knowledge that a few recipe cards will hardly prevent any type 2 diabetes case”. This comment was related to the criticism of intervention costs that was described in the opportunity costs section.

Regarding general reflections on effectiveness, several implementers (O3, O4, O11, O14) discussed the time needed for interventions to take effect. The implementers noted that changes rarely happen overnight, referring both to intervention implementation, which may require changes in organisational culture and practices, and to intervention impact, which requires readiness from the employees to adopt the intervention and to change own behaviour. Hence, to enhance adoption, one implementer (O14) suggested leveraging messengers that show the way and encourage colleagues to try out new things. This suggestion aligns with the observation on how the social work environment can enhance effectiveness. Further propositions included a digital app-assisted delivery besides print materials (O14) and the provision of intervention materials in English besides Finnish to consider employees with immigrant background (O8).

Concerning the persistence of intervention effects, the reports of several implementers (O2, O10, O11, O12) indicated that over time people may get numb to the intervention and initial effects may begin to fade. This remark applied to strategies that prompt suggested behaviours with attention-capturing cues and to strategies that require commitment and active participation. To sustain the effectiveness of attention-capturing prompts, one implementer (O2) suggested refreshing intervention materials and their placement occasionally. To encourage the continuation of commitment-requiring activities, the same implementer suggested minor rewards. For example, the employees might find more motivating to keep arranging fruit provision in coffee rooms if the employer occasionally organised the fruit service for them. This remark aligns with the above-mentioned observation that the employer’s financial support for the arrangement of healthy food provision at the worksite, either in the form of money, time, or facilities needed for implementation, appeared to accompany positive perceived effects.

### Employee perspective

In total 1124 employees from 15/16 participating organisations completed the questionnaire at the end of the intervention. The sample represents approximately 22% of the total number of employees who worked at the intervention sites. The mean response rate across organisations, including the one with zero respondents, was 31% (SD 23, range 0–68%). Of the respondents, 20% had a physical work, 29% used to eat at the worksite cafeteria, 37% wished that the employer would provide support for healthy eating, and 61% wished for support in physical activity.

Of all respondents, 95% considered acceptable that the employer seeks to influence the employees’ dietary and physical activity patterns to promote the employees’ wellbeing. The median overall acceptance of the specific choice architecture strategies evaluated was 7 (interquartile range IQR 6.4–7) (Table 6). The same applied to each specific strategy (Mdn 7, IQRs 6–7 to 7–7). Yet, we observed statistically significant differences between the distributions of acceptance of specific strategies (χ^2^(7) = 150.421, *p* < 0.001, *n* = 977). The level of acceptance of strategy (f.) that would improve the healthiness of foods and beverages available at the worksite—or in other words, replace less healthy options with healthier alternatives—was significantly lower compared to strategies that would (a.) provide information or tips on healthy eating and physical activity (*p* < 0.001), (c.) increase the relative availability of healthy options at the worksite cafeteria (*p* < 0.001), (d.) enhance the visibility and accessibility of healthy options at the worksite cafeteria (*p* = 0.018), (e.) clearly indicate healthy options at the worksite cafeteria (*p* = 0.005), and (g.) increase opportunities for physical activity at the worksite (*p* < 0.001). No significant differences were observed between any other strategies. Greater proportion of male employees at the intervention site was significantly associated with a lower overall acceptance score (OR 4.4, 95% CI 1.2 to 16.5) (Additional file 1). Physical work, eating at the worksite cafeteria, and wish for support in healthy eating or physical activity appeared unrelated with the acceptance. The proportion of opt-out responses (“I cannot say”) ranged from 1.3% to 7.6% across the strategies evaluated.

**Table 6:** Acceptance among employees of specific strategies that the employer would implement

Strategy	Behaviour change mechanism	n (%)^a^	Mdn^b^	IQR	Range	Opt-out, n (%)^c^
a. Information or tips related to healthy eating and physical activity distributed at the workplace	Provision of information	1103 (98.1)	7^b^	7–7	1–7	20 (1.8)
b. Reminders of wellbeing-promoting acts during working hours placed in the worksite environment	Point-of-choice prompt	1107 (98.5)	7^ab^	6–7	1–7	15 (1.3)
c. The proportion of healthy options increased at the worksite cafeteria supply	Availability	1040 (92.5)	7^b^	7–7	1–7	77 (6.9)
d. Healthy options placed on the most visible spots with the easiest access at the worksite cafeteria	Visibility, accessibility	1030 (91.6)	7^b^	7–7	1–7	85 (7.6)
e. Healthy options clearly marked at the worksite cafeteria	Provision of information, point-of-choice prompt	1032 (91.8)	7^b^	7–7	1–7	82 (7.3)
f. Foods and beverages served at the worksite made healthier, for example, at meetings or coffee breaks	Availability	1068 (95.0)	7^a^	6–7	1–7	49 (4.4)
g. Physically more active working enabled at the worksite, for example, with standing desks or exercise equipment for employees	Availability	1078 (95.9)	7^b^	7–7	1–7	40 (3.6)
h. Using the stairs instead of the elevator encouraged at the worksite, for example, with encouraging illustrations or markings that lead to the stairs	Point-of-choice prompt	1069 (95.1)	7^ab^	6–7	1–7	48 (4.3)
Overall acceptance score		977 (86.9)	7	6.4–7	1–7	na

^a^Number of numeric responses (% of total responses)

^b^Rating scale: 1 = completely disapprove, 7 = completely approve. Different superscript letters indicate statistically significant differences (*p*-value < 0.05) in pairwise comparisons

^c^Number of opt-out responses “I cannot say” (% of total responses). na = not applicable

## Discussion

This study evaluated the acceptability of a large-scale choice architecture intervention for healthy eating and daily physical activity at the workplace, considering the perspectives of implementers and influenced employees. The intervention applied a broad range of strategies, including primes, prompts, and alterations to the availability, visibility, and accessibility of choice options. Implementers considered the choice architecture approach ethical for workplace health promotion, expressed mostly positive affective attitudes to the intervention, and experienced little burden due to implementation. Intervention coherence supported acceptance through increased interest in implementation, whereas cost acceptance appeared dependent on the perceived utility and intensity of implementation. Perceived effectiveness was mixed. Employees expressed overall high acceptance of evaluated choice architecture strategies.

The support we observed for the choice architecture approach in workplace health promotion aligns with the results of population surveys that have demonstrated overall support for a range of choice architecture strategies implemented by various actors, including the employer [26], catering services [14, 21, 22], and policymakers [14–19, 24, 27]. The acceptance we observed might be partly explained by the intention of our intervention to promote small daily choices that contribute to the targeted individuals’ wellbeing and health. Populations across the globe appear to support choice architecture interventions perceived to have legitimate goals that serve the interests or values of most choosers [16, 17]. Relatedly, interventions intended to promote social good such as health have proved better accepted compared to interventions intended to increase the profits of the implementer [14, 23].

Another factor that may have contributed to the high acceptance of our intervention is the type of strategies implemented. Besides a few less transparent strategies in cafeterias and meetings, such as changed placement and portion sizes, most strategies and their intentions were transparent to the influenced employees. These transparent strategies either introduced new healthy choice options or cued the selection of such options with visual, attention-capturing cues that encouraged the promoted choices by making them attractive or salient, or by leveraging social norms and commitment. The dominance of these strategies in our intervention may be related to their applicability to diverse worksites regardless of resources, such as cafeterias or vending machines [39], or to their appeal to the designers who participated in their selection. The transparent strategies have been characterised as “epistemic transparent type 2 nudges”, or “empowerment nudges”, that engage automatic attention processes to facilitate reflected choices that individuals themselves evaluate as consistent with their preferences and interests [10]. While intentionally guiding people towards certain behaviours, these strategies promote autonomous decision-making and count as the least intrusive choice architecture interventions [10]. When disagreeing with the cues, people can easily and consciously neglect them. In prior acceptability evaluations, more transparent and less intrusive strategies such as nutrition labels have consistently received greater support compared to less transparent and more intrusive strategies, such as reductions to portion sizes or limitations to availability [15–21, 24, 26, 27]. Our employee-level data lent support for these findings. While the employees expressed high approval for all evaluated strategies, the data indicated that more intrusive strategies that replace less healthy foods with healthier alternatives may receive less support compared to less intrusive strategies that provide information or enhance the availability, visibility, or accessibility of healthier choices. Nevertheless, work communities and people in general appear to welcome the assistance that behavioural contexts can provide in overcoming the obesogenic influence of the contemporary living environment, which often translates to energy-dense and nutritionally poor food choices and sedentariness.

In terms of intervention coherence, our interview data indicated the importance of ensuring that implementers reach sufficient understanding of the purpose and working mechanism of applied intervention strategies. Such understanding could remain poor among implementers who did not participate in the designing phase of the intervention and whose role was to merely deliver the intervention. Relatedly, low perceived utility of the intervention was linked to poor approval of opportunity costs. Greater intervention coherence, in turn, not only promoted acceptability but appeared to enhance motivation for implementation as well. This observation supports the findings of our implementation evaluation [39] that demonstrated the importance of proper knowledge transfer to everyone involved in the implementation process, including those who miss the initial orientation and planning phase. Such knowledge sharing should help implementers to see the purpose and relevance of the intervention for themselves, their work community, and the organisation [39]. These insights provide empirical support for the Normalization Process Theory according to which the implementation, embedding, and integration of new practices in social contexts require that the practices are apprehended as meaningful, valuable, and useful [50].

Implementers expressed mostly positive affective attitudes to the content and implementation of the intervention, experienced overall little burden due to implementation, and rarely criticised costs; thus capturing the principle of choice architecture interventions being simple and inexpensive to implement [1, 42]. Yet, a small group of implementers criticised the content and costs of the intervention, as well as the burden related to engaging in the promoted behaviour. This criticism concerned particularly the packed lunch recipe campaign, which all sites intended to implement and which was the most extensively discussed intervention strategy. The critique applied to the type of recipes included in the campaign, the money spent on producing the materials (although the worksites received the materials free of charge), and the resources needed to deliver the materials. The criticism is understandable taken people’s varying values, food preferences, and resources for food preparation. People tend to agree with choice architecture interventions that meet their preferences and support needs [25, 26]. Yet, our employee-level data provided no evidence of an association between employees’ wish for support in healthy eating or physical activity and their overall approval of the evaluated strategies. Greater proportion of male employees per site, however, predicted lower acceptance; corroborating earlier evidence of a gender difference in the acceptance of choice architecture interventions [15–17, 19, 20, 24, 27].

An interesting feature of the received critique was that it often (though not always) came from implementers who were not involved in designing the intervention. While we tailored the content and implementation of the intervention to fit local contexts in collaboration with selected members of the personnel of the participating organisations, the personnel involved in the design process may have been insufficiently familiar with the employees of the intervention sites and hence unable to consider the hopes and needs of all employee groups. On the other hand, related to the above-discussed observations on intervention coherence, the implementers who missed the design process may have had poorer understanding of the purpose, rationale, and working mechanism of the intervention, which may have negatively influenced their attitudes to the intervention. In addition, the implementers behind the critique were mostly individuals whose work substance was unrelated to the promotion of employee wellbeing and health. Consequently, they might have been overall less interested in activities for nutrition and health. While these findings highlight the importance of designing publicly funded health-promotion interventions that acknowledge the target population’s preferences, they simultaneously demonstrate the difficulty of finding population-level strategies that appeal to everyone.

Although the implementers perceived many strategies to elicit positive effects, reports of negligible effects were also common. Factors accompanying positive effects involved an active implementer, supportive social and physical work environment, and employer-granted financial support for implementation. Besides supporting the target audience in engaging in the promoted behaviour, these factors facilitate implementation [39], which in turn predicts greater effectiveness [51, 52]. In terms of perceived ineffectiveness, the explanations our implementers suggested included varying individual preferences, needs, and understanding of the intervention. The suggestions relate to the discussed relationship between preferences and affective attitudes to the intervention and receive support from prior choice architecture research in which conflicts between the intervention and the target group’s preferences have proved barriers to intervention effectiveness [11, 53].

Another potential explanation to the varying perceived effectiveness is the type of intervention strategies employed. As mentioned, the most frequently implemented strategies in our intervention count as so-called epistemic transparent type 2 nudges [10]—also known as cognitively oriented nudges [54]—that promote reflected choices. While such strategies are the least intrusive and appear best accepted within the choice architecture approach [15–21, 24, 26, 27], their effect sizes tend to be small [54, 55]. Yet, anticipated and true effectiveness of choice architecture strategies seem inversely correlated [19]. This misconception may have contributed to our designers’ proneness to select strategies that yield relatively small effects.

In our implementer reports, perceived effectiveness was linked with affective attitudes and views on opportunity costs. More specifically, perceived effectiveness could influence the implementers’ interest in sustaining the intervention and their approval of the resources that were invested in the intervention. These observations are analogous to our findings on factors that facilitate implementation [39] and support prior research that has found perceived effectiveness an important predictor of acceptability [14, 15, 18, 19]. Yet, we remind that perceived effectiveness may deviate from true effectiveness [19] and can depend on, for example, received information on expected impact [15] or personal experiences of intervention effects [14]. Hence, perceived effectiveness mainly reflects the implementers’ attitudes to the usefulness of the intervention [23].

### Strengths and limitations

The strengths of this study include the theory-based, multidimensional acceptability evaluation of a broad range of choice architecture strategies that were selected for implementation in collaboration with participating organisations and integrated into the daily operations of heterogeneous worksites. The evaluation covered the perspectives of two key groups within work communities, implementers and influenced employees, finding both groups to support the choice architecture approach for promoting healthy eating and daily physical activity at the workplace. The implementers included both individuals who had participated in designing the intervention to their worksites and individuals who had not. Regarding the implementers, the evaluation covered experienced (i.e., concurrent and retrospective) acceptability of the intervention and its implementation, acknowledging the multi-faceted definition of acceptability. The evaluation drew a nuanced view of the multitude of factors that influence acceptance and consequently implementation and effectiveness, providing support for the development of improved interventions [12, 13]. The study stretches beyond prior research that has mainly evaluated anticipated (i.e., prospective) acceptability of hypothetical choice architecture interventions among potential target audiences [14–27]. Regarding employees, our evaluation covered the retrospective evaluation of eight specific intervention strategies employed in the intervention. In this respect, the work adds to the few existing choice architecture studies that have examined the influenced individuals’ experienced acceptance in the real world [28–31]. Moreover, with rich data from the field, the present study contributes to the translation and upscaling of choice architecture interventions from controlled behavioural laboratories and living labs to diverse real-world settings, providing insights on the feasibility of various choice architecture strategies in the workplace context.

The study has its limitations as well. The strategies most frequently implemented in the intervention either introduced new healthy choice options or prompted healthy choices with attention-capturing visual cues. Such strategies represent the least intrusive choice architecture interventions that leave the freedom of choice fully to the targeted individuals. Hence, our results largely reflect the acceptability of the gentlest nudges. In addition, since the participating worksites implemented several intervention strategies simultaneously, the implementer-level analysis was unable to evaluate the acceptability of each individual strategy. Yet, where feasible, we indicated the specific strategies to which our implementers referred. Another limitation of the implementer-level assessment is that our implementers’ interview reports reflected to some extent a dual perspective, that of the intervention deliverer and that of the intervention receiver, and in certain domains, these two perspectives were impossible to distinguish. The reason for this mixing was that the implementers were selected among the personnel of the intervention sites. Consequently and unavoidably, similar to other employees at their sites, the implementers too became exposed to and influenced by the intervention. The positive side of this dual perspective is that the implementer-level data partly complements the employee-level data. As for the employee-level analysis, due to privacy protection, our questionnaire did not collect identifiable data on individual respondents. We were hence unable to examine the extent to which our sample represents the employee population across the participating organisations, and whether individual characteristics such socio-economic background influence acceptance.

### Implications for practice and research

Our empirical findings suggest that from the perspective of acceptability, workplaces can safely adopt the choice architecture approach as a tool to create worksite environments that support the personnel in adopting and maintaining healthy lifestyles. For a broad acceptance within the work community, including both implementers and influenced employees, we recommend involving representative members of each personnel group in designing intervention content and implementation, acknowledging the factors this study identified to influence acceptance. Particularly, we recommend ensuring sufficient understanding of the intervention among implementers, and tailoring intervention content to the personnel’s needs, values, and preferences as far as possible within a group-level intervention. Future studies could evaluate the acceptability of more intrusive choice architecture strategies for promoting healthy eating and daily physical activity at the workplace, for example, setting healthy options the default choices. Additionally, studies could compare the acceptance of choice architecture interventions with other types of workplace interventions for healthy eating and daily physical activity, for example, limitations to the availability of unhealthy options at the worksite, knowledge-based lifestyle coaching programs, and financial (dis)incentives for (un)healthy choices. Regarding the perspective of influenced employees, collecting demographic data on individual respondents would enable the comparison of acceptance between diverse employee groups.

## Conclusions

This acceptability evaluation of a large-scale choice architecture intervention for healthy eating and daily physical activity at the workplace found a broad range of choice architecture strategies overall acceptable for workplace health promotion, yet identified numerous facilitators and barriers of acceptance. The work adds to prior research with a theory-based analysis that considered multiple dimensions of acceptability and included the perspectives of two key groups within work communities, implementers and influenced employees, once they had experienced the intervention. The work provides insights on the upscaling of choice architecture interventions to heterogeneous real-world settings and supports the development of improved interventions.

### Supplementary Information

**Additional file 1.** 

## Data Availability

The datasets used and/or analysed during the current study, including quantitative data and qualitative Finnish language data, are available from the corresponding author on a reasonable request.

## References

[1] Thaler RH, Sunstein CR (2009). Nudge: Improving decisions about health, wealth, and happiness.

[2] Hansen PG (2016). The definition of nudge and libertarian paternalism: Does the hand fit the glove?. European Journal of Risk Regulation.

[3] Marteau TM, Fletcher PC, Hollands GJ, Munafò MR, Hagger M, Cameron LD, Hamilton K, Hankonen N, Lintunen T (2020). Changing Behavior by Changing Environments. The Handbook of Behavior Change.

[4] Deutsch R, Strack F, Hagger MS, Cameron LD, Hamilton K, Hankonen N, Lintunen T (2020). Changing Behavior Using the Reflective-Impulsive Model. The Handbook of Behavior Change.

[5] Kahneman D (2003). Maps of bounded rationality: Psychology for behavioral economics. Am Econ Rev.

[6] OECD, EU. Health at a Glance: Europe 2016 - State of Health in the EU Cycle. Paris: OECD Publishings; 2016.

[7] Krekel C, Ward G, De Neve J-E. Employee Wellbeing, Productivity, and Firm Performance. Saïd Business School WP 2019–04. 2019. 10.2139/ssrn.3356581.

[8] Ensaff H (2021). A nudge in the right direction: the role of food choice architecture in changing populations’ diets. Proc Nutr Soc.

[9] Hollands GJ, Marteau TM, Fletcher PC (2016). Non-conscious processes in changing health-related behaviour: A conceptual analysis and framework. Health Psychol Rev.

[10] Hansen PG, Jespersen AM (2013). Nudge and the manipulation of choice: A framework for the responsible use of the nudge approach to behaviour change in public policy. Eur J Risk Reg.

[11] de Ridder D, Kroese F, van Gestel L (2022). Nudgeability: Mapping Conditions of Susceptibility to Nudge Influence. Perspect Psychol Sci.

[12] Sekhon M, Cartwright M, Francis JJ (2018). Acceptability of health care interventions: A theoretical framework and proposed research agenda. Br J Health Psychol.

[13] Sekhon M, Cartwright M, Francis JJ (2017). Acceptability of healthcare interventions: an overview of reviews and development of a theoretical framework. BMC Health Serv Res.

[14] Bang HM, Shu SB, Weber EU (2020). The role of perceived effectiveness on the acceptability of choice architecture. Behav Public Policy.

[15] Reynolds JP, Archer S, Pilling M, Kenny M, Hollands GJ, Marteau TM (2019). Public acceptability of nudging and taxing to reduce consumption of alcohol, tobacco, and food: A population-based survey experiment. Soc Sci Med.

[16] Sunstein CR, Reisch LA, Rauber J (2018). A worldwide consensus on nudging? Not quite, but almost. Regul Govern.

[17] Reisch LA, Sunstein CR (2016). Do Europeans Like Nudges?. Judgm Decis Mak.

[18] Petrescu DC, Hollands GJ, Couturier D-L, Ng Y-L, Marteau TM (2016). Public Acceptability in the UK and USA of Nudging to Reduce Obesity: The Example of Reducing Sugar-Sweetened Beverages Consumption. PLoS ONE.

[19] Cadario R, Chandon P (2019). Viewpoint: Effectiveness or consumer acceptance? Tradeoffs in selecting healthy eating nudges. Food Policy.

[20] Evers C, Marchiori DR, Junghans AF, Cremers J, De Ridder DTD (2018). Citizen approval of nudging interventions promoting healthy eating: the role of intrusiveness and trustworthiness. BMC Public Health.

[21] Nørnberg TR, Skov LR, Houlby L, Pérez-Cueto FJA (2016). Attitudes and Acceptability of Behavior Change Techniques to Promote Healthy Food Choices Among Danish Adolescents. Fam Consum Sci Res J.

[22] dos Santos Q, Perez-Cueto FJA, Rodrigues VM, Appleton K, Giboreau A, Saulais L (2020). Impact of a nudging intervention and factors associated with vegetable dish choice among European adolescents. Eur J Nutr.

[23] Junghans AF, Cheung TT, De Ridder DD (2015). Under consumers’ scrutiny - an investigation into consumers’ attitudes and concerns about nudging in the realm of health behavior. BMC Public Health.

[24] Hagmann D, Siegrist M, Hartmann C (2018). Taxes, labels, or nudges? Public acceptance of various interventions designed to reduce sugar intake. Food Policy.

[25] Harbers MC, Middel CNH, Stuber JM, Beulens JWJ, Rutters F, van der Schouw YT (2021). Determinants of Food Choice and Perceptions of Supermarket-Based Nudging Interventions among Adults with Low Socioeconomic Position: The SUPREME NUDGE Project. Int J Environ Res Public Health.

[26] Felsen G, Castelo N, Reiner PB (2013). Decisional enhancement and autonomy: public attitudes towards overt and covert nudges. Judgm Decis Mak.

[27] Dieteren CM, Bonfrer I, Brouwer WBF, Van Exel J (2023). Public preferences for policies promoting a healthy diet: a discrete choice experiment. Eur J Health Econ.

[28] Van Gestel LC, Kroese FM, De Ridder DTD (2018). Nudging at the checkout counter–A longitudinal study of the effect of a food repositioning nudge on healthy food choice. Psychol Health.

[29] Kroese FM, Marchiori DR, de Ridder DTD (2016). Nudging healthy food choices: a field experiment at the train station. J Public Health.

[30] Villinger K, Wahl DR, Engel K, Renner B (2021). Nudging sugar portions: a real-world experiment. BMC Nutrition.

[31] Hansen PG, Schilling M, Malthesen MS (2021). Nudging healthy and sustainable food choices: three randomized controlled field experiments using a vegetarian lunch-default as a normative signal. J Public Health.

[32] Timm L, Annerstedt KS, Ahlgren JÁ, Absetz P, Alvesson HM, Forsberg BC (2022). Application of the Theoretical Framework of Acceptability to assess a telephone-facilitated health coaching intervention for the prevention and management of type 2 diabetes. PLoS ONE.

[33] Laitinen AL, Antikainen A, Mikkonen S, Kähkönen K, Talvia S, Varjonen S (2023). The ‘Tasty School’ model is feasible for food education in primary schools. J Human Nutrition Diet.

[34] De Groot LM, Shearer K, Sambani C, Kaonga E, Nyirenda R, Mbendera K, et al. Health care providers acceptance of default prescribing of TB preventive treatment for people living with HIV in Malawi: a qualitative study. preprint. In Review; 2023. 10.21203/rs.3.rs-3148655/v1. 10.1186/s12913-023-10493-9 PMC10768226 38178173

[35] Perez-Cueto FJA (2021). Nudging plant-based meals through the menu. Int J Gastronomy Food Sci.

[36] Saulais L, Massey C, Perez-Cueto FJA, Appleton KM, Dinnella C, Monteleone E (2019). When are “Dish of the Day” nudges most effective to increase vegetable selection?. Food Policy.

[37] Hartwell H, Bray J, Lavrushkina N, Rodrigues V, Saulais L, Giboreau A (2020). Increasing vegetable consumption out-of-home: VeggiEAT and Veg+projects. Nutr Bull.

[38] Landes SJ, McBain SA, Curran GM (2019). An introduction to effectiveness-implementation hybrid designs. Psychiatry Res.

[39] Rantala E, Vanhatalo S, Tilles-Tirkkonen T, Kanerva M, Hansen PG, Kolehmainen M (2021). Choice Architecture Cueing to Healthier Dietary Choices and Physical Activity at the Workplace: Implementation and Feasibility Evaluation. Nutrients.

[40] Pihlajamäki J, Männikkö R, Tilles-Tirkkonen T, Karhunen L, Kolehmainen M, Schwab U (2019). Digitally supported program for type 2 diabetes risk identification and risk reduction in real-world setting: protocol for the StopDia model and randomized controlled trial. BMC Public Health.

[41] Lakka TA, Aittola K, Järvelä-Reijonen E, Tilles-Tirkkonen T, Männikkö R, Lintu N (2022). Real-world effectiveness of digital and group-based lifestyle interventions as compared with usual care to reduce type 2 diabetes risk – A stop diabetes pragmatic randomised trial. Lancet Reg HealthEur..

[42] Hollands GJ, Bignardi G, Johnston M, Kelly MP, Ogilvie D, Petticrew M (2017). The TIPPME intervention typology for changing environments to change behaviour. Nat Hum Behav.

[43] Dolan P, Hallsworth M, Halpern D, King D, Metcalfe R, Vlaev I (2012). Influencing behaviour: The mindspace way. J Econ Psychol.

[44] Hallsworth M, Halpern D, Algate F, Gallagher R, Nguyen S, Service O (2016). EAST - Four simple ways to apply behavioural insights.

[45] The Finnish Heart Association. Heart Symbol. 2023. https://www.sydanmerkki.fi/en/. Accessed 3 Dec 2023.

[46] Tong A, Sainsbury P, Craig J (2007). Consolidated criteria for reporting qualitative research (COREQ): a 32-item checklist for interviews and focus groups. Int J Qual Health Care.

[47] Elo S, Kyngäs H (2008). The qualitative content analysis process. J Adv Nurs.

[48] Ng A, Reddy M, Zalta AK, Schueller SM (2018). Veterans’ Perspectives on Fitbit Use in Treatment for Post-Traumatic Stress Disorder: An Interview Study. JMIR Mental Health.

[49] Borghouts J, Eikey E, Mark G, De Leon C, Schueller SM, Schneider M (2021). Barriers to and Facilitators of User Engagement With Digital Mental Health Interventions: Systematic Review. J Med Internet Res.

[50] May C, Finch T (2009). Implementing, Embedding, and Integrating Practices: An Outline of Normalization Process Theory. Sociology.

[51] Wierenga D, Engbers LH, Van Empelen P, Duijts S, Hildebrandt VH, Van Mechelen W (2013). What is actually measured in process evaluations for worksite health promotion programs: A systematic review. BMC Public Health.

[52] Durlak JA, DuPre EP (2008). Implementation matters: A review of research on the influence of implementation on program outcomes and the factors affecting implementation. Am J Community Psychol.

[53] Rantala E, Järvelä-Reijonen E, Pettersson K, Laine J, Vartiainen P, Närväinen J (2022). Sensory appeal and routines beat health messages and visibility enhancements: mixed-methods analysis of a choice-architecture intervention in a workplace cafeteria. Nutrients.

[54] Cadario R, Chandon P (2020). Which Healthy Eating Nudges Work Best? A Meta-Analysis of Field Experiments. Mark Sci.

[55] Mertens S, Herberz M, Hahnel UJJ, Brosch T (2022). The effectiveness of nudging: A meta-analysis of choice architecture interventions across behavioral domains. Proc Natl Acad Sci USA.

