# Supplementary material for: Acceptability of workplace choice architecture modification for healthy behaviours
Source: BMC Public Health. 2023 Dec 7;23:2451. doi: 10.1186/s12889-023-17331-x (PMC10704714; doi:10.1186/s12889-023-17331-x)
Supplement: Supplementary file 1 — Additional file 1. [file 12889_2023_17331_MOESM1_ESM.pdf]

Supplementary material

Acceptability of workplace choice architecture modification for healthy behaviours

Data collection

Acceptability-related questions of implementer interviews translated from Finnish to English

First interview halfway through the intervention

1. What intervention strategies did you implement?
2. How did the launch of the intervention go?
3. How has the sustaining of the intervention gone?
4. What has worked well in the implementation? What factors have contributed to these successes?
5. Have there been difficulties in the implementation? If so, what kind of difficulties have there been and how have the difficulties been resolved?
6. How could the implementation be promoted at your workplace? What would it take?
7. What has motivated you in the implementation? Has something been unmotivating?
8. Have you presented the intervention materials to the employees or encouraged the employees to use the materials?
9. Are you the most appropriate person in your organisation to take care of the implementation, or would someone else be more appropriate?
10. How has the intervention been received? Have the employees noticed or discussed the intervention? Have you heard any feedback?
11. What kind of effects have you observed? Have the intervention materials been used? Have you noticed changes in the employees' behaviour?
12. Do you find it acceptable that the employer attempts to influence the employees' health behaviour?
13. In your opinion, in what ways is the employer allowed to aim at influencing the employees' health behaviour?
14. Do you find choice architecture interventions an acceptable approach to promote healthy dietary choices and physical activity among employees? Choice architecture interventions mean modifying the work environment in such a way that it gently guides employees to health-promoting habits.

Second interview at the end of the intervention

1. Has anything changed in the implementation after the 6-month follow-up? For example, the schedule of completing implementation-related tasks; informing the employees of intervention materials or promoting the materials to the employees.
2. How has the intervention been received? Have the employees noticed or discussed the intervention? Have you heard any feedback?
3. What kind of effects have you observed? Have the intervention materials been used? Have you noticed changes in the employees' behaviour?

Statistical analyses

Mixed-effects logistic regression model examined the association between the employees' overall (i.e., mean) acceptance of eight specific choice architecture strategies (dependent variable) and five site-level predictors (independent variables). For the model, the overall acceptance score was transformed into a dichotomous variable, with scores below the 25th percentile at 6.38 treated as the target category (n=230) and scores at or above the 25th percentile as the reference category (n=747). The site-level predictors included in the model were: (1) the proportion of male employees at the site during the intervention year, (2) the proportion of respondents with physical work, (3) the proportion of respondents with a habit of eating at the worksite cafeteria, (4) the proportion of respondents who wished that the employer would provide support for healthy eating, and (5) the proportion of respondents who wished that the employer would provide support for physical activity. The model was specified with a 2-level data structure using intervention worksite (or organisation if the questionnaire data was collected at the level of the participating organisation) as the clustering variable. The model was built with the generalised linear mixed model (GENLINMIXED) routine of IBM SPSS statistics® version 29.0 (IBM Corp., Armonk, NY, USA). In GENLINMIXED, the default estimation method is a quasilikelihood approach called active set method (ASM) with Newton-Raphson estimation (Heck et al., 2012, p. 27). We included random intercept as the random effect and selected variance components as the covariance structure for the random coefficients. We selected the Satterthwaite

approximation to the degrees of freedom that were used to compute significance tests for model parameters, as recommended for data with varying number of individuals across clusters (Heck et al., 2012, p. 147). Additionally, we selected a robust, more conservative approach to the calculation of the standard errors of regression coefficients to allow departures from normality.

The predictors included in the model were summarised to the site-level and grand-mean centred within the dataset that was included in the analysis by subtracting the overall sample mean from the site-level value. Grand-mean-centring recentres the site's standing on the variable against the sample mean and facilitates the interpretation of the coefficients of model parameters (Heck et al., 2012, p. 21). Summarising to the site level was necessary for the following dichotomous variables that were measured at the individual level: physical work, a habit of eating at the worksite cafeteria, and wish for support in healthy eating/physical activity. The summarising involved computing the proportion of individuals per site with the desired characteristic (e.g., physical work), and assigning the resulting values to the individual respondents of the corresponding site.

Results

Table S1. Associations between site-level predictors and an overall acceptance score below the 25th percentile (n=977).

Predictors included in the model	OR (95% CI)[1]	p-value[1]
Male employees per site	4.4 (1.2; 16.5)	.033
Respondents with physical work	2.2 (0.3; 15.5)	.388
Respondents eating at the worksite cafeteria	0.9 (0.1; 7.6)	.919
Respondents hoping for support in healthy eating	0.3 (0.0; 5.7)	.391
Respondents hoping for support in physical activity	1.2 (0.0: 128.3)	.930

[1] Odds ratio (95% confidence interval) and the significance of association between each predictor and an overall acceptance score below the 25th percentile, controlling for all the other predictors in the mixed-effects logistic regression model.

Reference

Heck, R. H., Thomas, S. L., & Tabata, L. N. (2012). Multilevel modeling of categorical outcomes using IBM SPSS. Routledge.
